# Supplementary material for: Tensor‐structured decomposition improves systems serology analysis
Source: Mol Syst Biol. 2021 Sep 6;17(9):e10243. doi: 10.15252/msb.202110243 (PMC8420856; doi:10.15252/msb.202110243)
Supplement: Supplementary file 2 — Expanded View Figures PDF [file MSB-17-e10243-s002.pdf]

Expanded View Figures

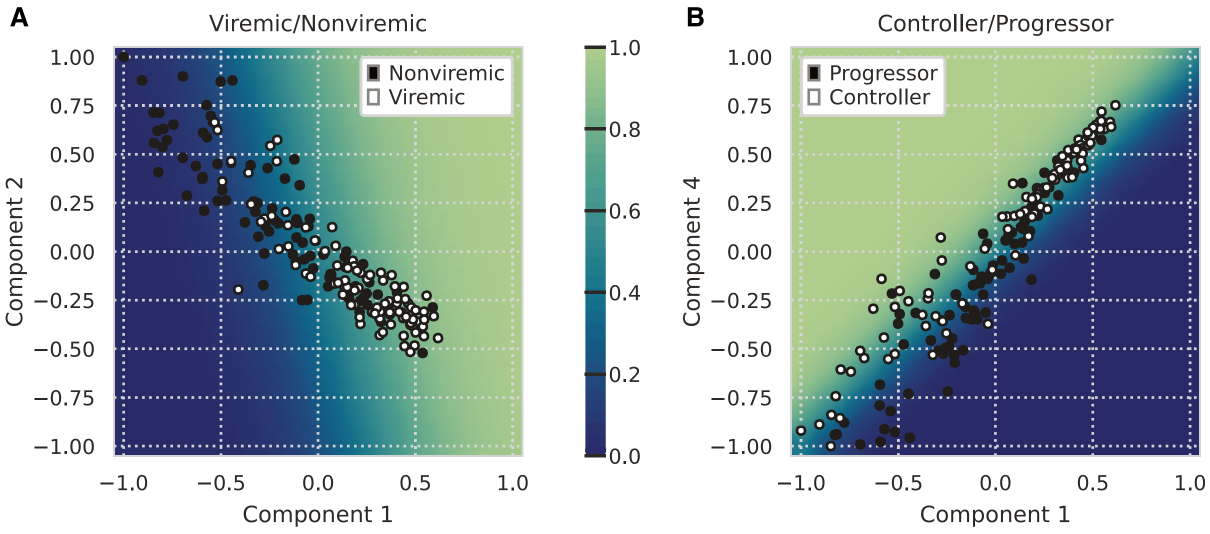

Figure EV1. Decision boundaries of subject classification.

A Viremic/non-viremic decision boundary, plotted as the probability of being viremic.

B Controller/progressor decision boundary, plotted as the probability of being a controller.

© 2021 The Authors

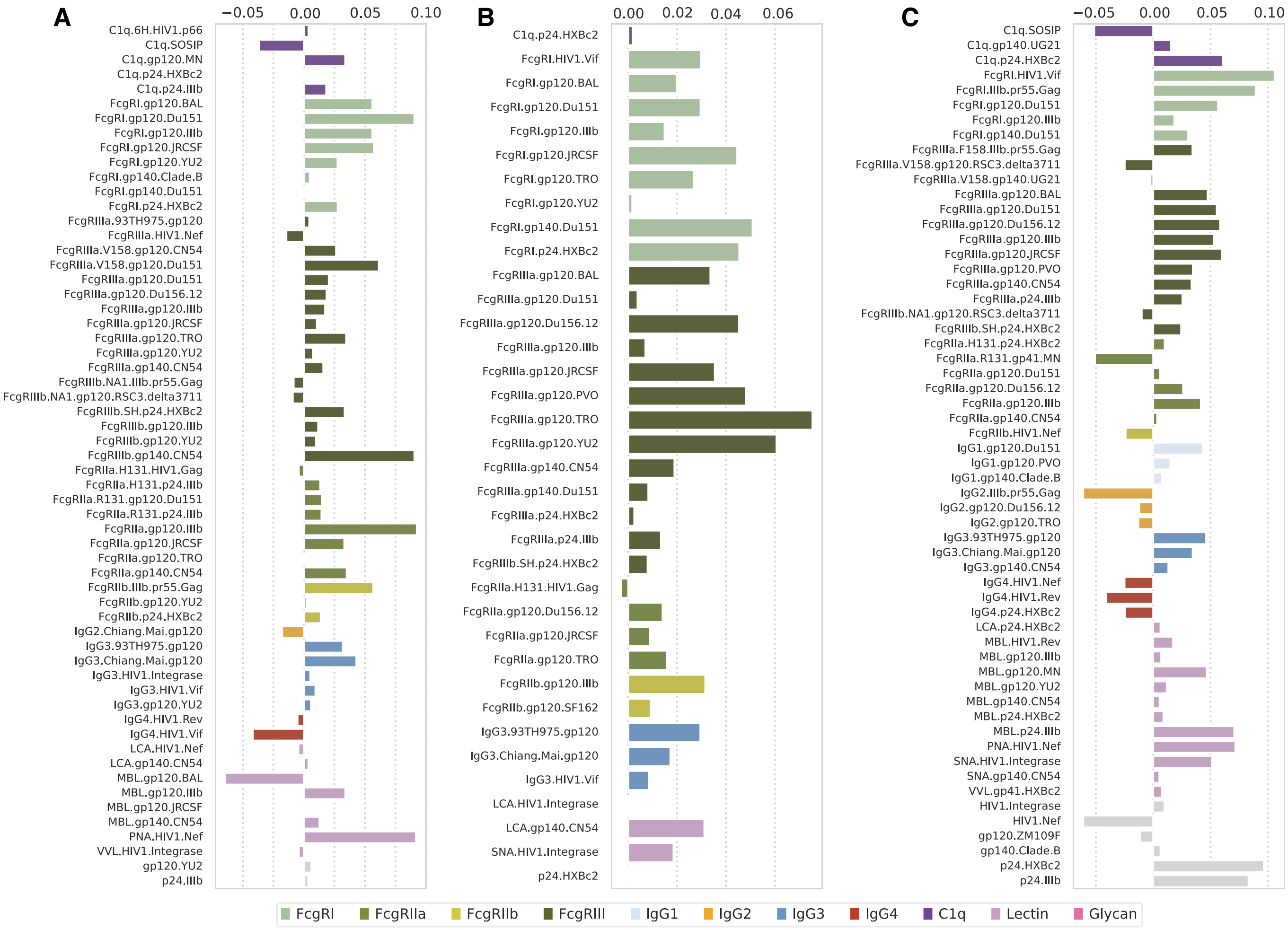

Figure EV2. Three runs of bootstrapped samples of elastic net model predicting ADCD.

A–C Each subplot shows the feature weights from an independent run of the elastic net model with bootstrapping predicting ADCD. Bars are colored according to the category of each input variable. Bootstrapping was performed by resampling subjects with replacement and then rebuilding the elastic net model predicting ADCD.

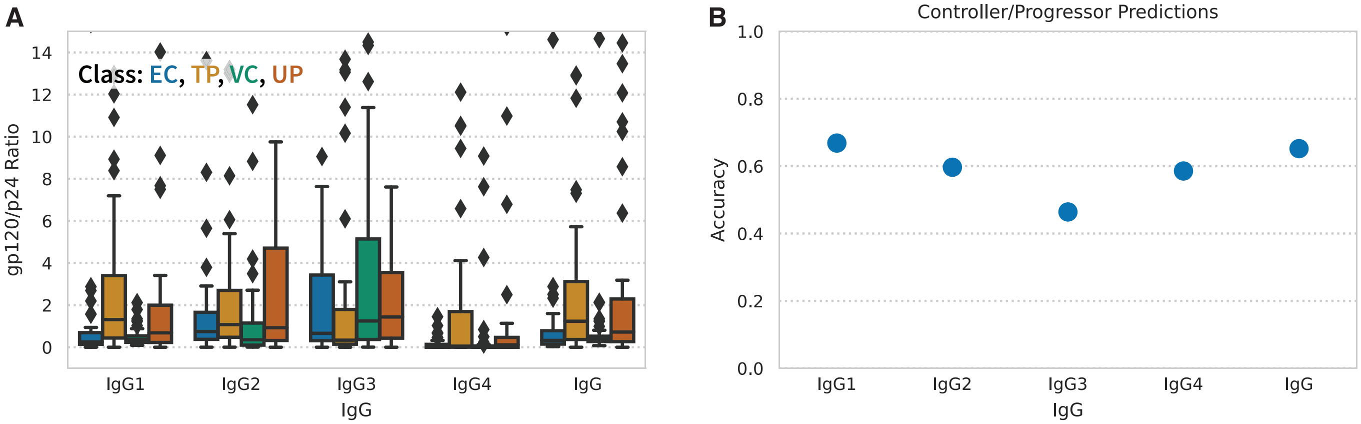

Figure EV3.

 © 2021 The Authors

Figure EV3. A simple gp120/p24 antigen ratio predicts progression to varying degrees.

A Raw gp120/p24 IgG measurement ratios, separated by subject class. Data plotted as boxplots. Each point represents a distinct subject (EC $N = 45$, TP $N = 44$, VC $N = 51$, UP $N = 41$). The three bands in each box represent the first, second, and third quartiles of the ratios, from the bottom to the top, respectively; the whiskers extend up to 1.5 times the interquartile range beyond the box range; any outliers beyond the whisker ends are plotted as single points.

B Controller/progressor prediction accuracy using gp120/p24 ratios of each IgG isotype. Predictions were performed using logistic regression as described in methods. Accuracy is defined as the percent correctly classified.
